# Crohn’s Disease Phenotypes and Associations With Comorbidities, Surgery Risk, Medications and Nonmedication Approaches: The MAGIC in IMAGINE Study

**DOI:** 10.1093/ibd/izae055

**Published:** 2024-03-27

**Authors:** Charles N Bernstein, Remo Panaccione, Zoann Nugent, Deborah A Marshall, Gilaad G Kaplan, Stephen Vanner, Levinus A Dieleman, Lesley A Graff, Anthony Otley, Jennifer Jones, Michelle Buresi, Sanjay Murthy, Mark Borgaonkar, Brian Bressler, Alain Bitton, Kenneth Croitoru, Sacha Sidani, Aida Fernandes, Paul Moayyedi

**Affiliations:** Department of Internal Medicine, Max Rady College of Medicine, Rady Faculty of Health Sciences, University of Manitoba, Winnipeg, Manitoba, Canada; University of Manitoba Inflammatory Bowel Disease Clinical and Research Centre, Winnipeg, Canada; Division of Gastroenterology and Hepatology, Departments of Medicine, University of Calgary, Calgary, Alberta, Canada; Department of Internal Medicine, Max Rady College of Medicine, Rady Faculty of Health Sciences, University of Manitoba, Winnipeg, Manitoba, Canada; University of Manitoba Inflammatory Bowel Disease Clinical and Research Centre, Winnipeg, Canada; Departments of Community Health Sciences, University of Calgary, Calgary, Alberta, Canada; Division of Gastroenterology and Hepatology, Departments of Medicine, University of Calgary, Calgary, Alberta, Canada; Departments of Community Health Sciences, University of Calgary, Calgary, Alberta, Canada; Queens University, Kingston, Ontario, Canada; Department of Medicine, University of Alberta, Edmonton, Alberta, Canada; University of Manitoba Inflammatory Bowel Disease Clinical and Research Centre, Winnipeg, Canada; Department of Clinical Health Psychology, Max Rady College of Medicine, Rady Faculty of Health Sciences, University of Manitoba, Winnipeg, Manitoba, Canada; Department of Pediatrics, Dalhousie University, Halifax, Nova Scotia, Canada; Department of Internal Medicine, Dalhousie University, Halifax, Nova Scotia, Canada; Division of Gastroenterology and Hepatology, Departments of Medicine, University of Calgary, Calgary, Alberta, Canada; Department of Medicine, University of Ottawa, Ottawa, Ontario, Canada; Department of Medicine, Memorial University, St Johns, Newfoundland, Canada; Department of Medicine, University of British Columbia, Vancouver, BC, Canada; Department of Medicine, McGill University, Montreal, Quebec, Canada; Department of Medicine, University of Toronto, Toronto, Ontario, Canada; Department of Medicine, University of Montreal, Montreal, Quebec, Canada; Department of Medicine, McMaster University, Hamilton, Ontario, Canada; Department of Medicine, McMaster University, Hamilton, Ontario, Canada

**Keywords:** phenotype, cohort study, Crohn’s disease, surgery

## Abstract

**Background:**

We aimed to establish a cohort of persons with Crohn’s disease (CD) enrolled from 14 Canadian centers to describe the contemporary presentation of CD in Canada.

**Methods:**

All enrollees were at least 18 years old and underwent chart review for phenotype documentation by Montreal Classification at time of enrollment, comorbidities, inflammatory bowel disease (IBD) and other surgeries, and use IBD and other therapies.

**Results:**

Of 2112 adults, 59% were female, and the mean age was 44.1 (+/-14.9SD) years. The phenotype distribution was B1 = 50.4%, B2 = 22.4%, B3 = 17.3%, and missing information = 9.9%. Perineal disease was present in 14.2%. Pertaining to disease location, 35.2% of patients had disease in L1, 16.8% in L2, 48% in L3, and 0.4% in L4. There was no difference in phenotype by gender, anxiety score, depression score. Disease duration was significantly different depending on disease behavior type (B1 = 12.2 ± 10.1; B2 = 19.4 ± 12.9; B3 = 18.9 ± 11.8, *P* < .0001). Isolated colonic disease was much less likely to be fibrostenotic or penetrating than inflammatory disease. Penetrating disease was more likely to be associated with ileocolonic location than other locations. Perineal disease was most commonly seen in persons with B3 disease behavior (24%) than other behaviors (11% B1; 20% B2 disease, *P* < .0001) and more likely to be seen in ileocolonic disease (L3;19%) vs L2 (17%) and L1 (11%; *P* < .0001). Surgery related to IBD occurred across each behavior types at the following rates: B1 = 23%, B2 = 64%, and B3 = 74%. Inflammatory bowel disease–related surgery rates by location of disease were L1 = 48%, L2 = 21%, and L3 = 51%.

**Conclusions:**

In exploring this large contemporary CD cohort we have determined that inflammatory disease is the main CD phenotype in Canada and that CD-related surgery remains very common.

**Key Messages**

**What is already known?**

There are several large cohort studies from across the world that suggest that over time most persons with Crohn’s disease (CD) will have complicated disease manifested by either fibrostenosis or penetrating disease.

**What is new here?**

The most common CD behavior type among a large cohort adult Canadians is inflammatory after a mean of 15 years’ disease duration. Approximately 2 of every 5 persons with CD underwent CD-related surgery.

**How can this study help patient care?**

This study of one of the largest contemporary cohorts of adults with CD reminds clinicians of the most common patterns of disease to be encountered, the ongoing common use of corticosteroids and 5-aminosalicylates as treatment, and the ongoing need for CD-related surgery in a biologic era.

## Introduction

While previous research has explored the phenotypic expression of Crohn’s disease (CD) in different populations,^1–10^ there is a lack of studies reporting on the association between CD phenotypes and common comorbidities, or the range of interventions including surgeries, medication, diets, and complementary therapy use. Perhaps clues to phenotypic differentiation and more comprehensive subclassification of CD can emerge if certain subtypes associate with these aspects more specifically. Further, there has been a long-held consideration that the majority of persons with inflammatory CD will progress over time to complicated disease (fibrostenosing and/or penetrating), and it is of interest to determine what modifiable factors, including treatment approaches, contribute to prediction of these trajectories.^1,2^

Several studies have reported on risk factors associated with different CD phenotypes. One research study correlated prediagnosis antimicrobial serotypes with presenting phenotype.^11^ A multicenter Canadian pediatric study reported on variation in phenotype of IBD throughout the pediatric age spectrum.^12^ A study from the Swiss patient registry reported on genetic predictors of some phenotypes.^13^ A study from India and Israel, with very distinct patient populations from a demographic perspective, reported that persons with newly diagnosed CD had distinct phenotypes comparing one country to the other.^14^

The data are mixed as to whether CD is predominantly inflammatory at baseline, and over time the phenotype becomes predominately complicated (fibrostenosing or penetrating). In a review comparing phenotype at baseline diagnosis to that at 10 years, there was very little change in disease location at 10 years in both Asian and western studies.^9^ However, there was a considerable spectrum regarding disease behavior in Asian and western populations. In Asian countries, approximately 30% have fibrostenosing or penetrating disease at baseline; and by 10 years, approximately 50% have complicated disease, indicating a change of approximately 20% over that time period.^9^ In a recent study from Japan of 673 newly diagnosed adults with CD, 63% had an inflammatory phenotype; 60% had ileocolonic disease, and 49% had perianal lesions, reflecting a very high rate of perineal disease, with the majority being perianal abscesses or fistulas.^10^ In other Asian studies, rates of inflammatory disease were even higher at 66% to 81%.^7–9^

In other western populations, the spectrum of disease behavior is more variable.^3–6^ Baseline rates of complicated disease were approximately 18% in Olmsted County (MN, USA), 27% in New Zealand, and 40% in Hungary and Norway, with increases by 10 years to 43% (7), 56% (5), 53% (6) and 72%, respectively.^3^ However, in a large French study (*n* = 1199), approximately 50% had complicated disease at diagnosis. By 20 years’ disease duration, the actuarial rate of having inflammatory disease was only 12%, as disease progression was so high that 88% had complicated disease, of which 70% had penetrating disease.^2^ This French study appears to be an outlier relative to the multiple other studies with more moderate rates of progression, but it has been often touted as a reason to aggressively treat CD early before complications emerge. In neighboring Belgium, 27% had complicated disease at baseline, and nearly 70% had complicated disease by 10 years.^1^ The phenotype of disease may impact on the need for CD-specific surgery, and the phenotype may be associated with other non-IBD surgeries, comorbidities, medication approaches, or even dietary choices.

The Inflammation, Microbiome, and Alimentation: Gastro-Intestinal and Neuropsychiatric Effects (IMAGINE) Strategy for Patient Oriented Research (SPOR) Chronic Disease Network is conducting a 5-year multicenter prospective observational cohort study, Mind And Gut Interactions Cohort (MAGIC) in 14 centers across Canada spanning 2018 to 2023.^15^ It is exploring the interactions across diet, microbiome composition, and the individual host associated with a diagnosis of IBD. Using the data from the MAGIC study in this analysis, we aim to determine the phenotypic distribution of a large well-characterized contemporary cohort of adult Canadians with CD and the relationship between CD phenotypes and reported comorbidities and a variety of common surgeries. We also report on predictors for CD-related surgery. Finally, we report on the diets, medications, and complementary and alternative treatments (CATs) used in contemporary management of CD in Canada and their relative distribution of use across the different phenotypes.

## Methods

For this cohort analysis limited to adults with Crohn’s disease, individuals had to be 18 years and older, have a definitive diagnosis of CD, confirmation of phenotype, and clinical histories through chart review, and be able to provide informed consent. While dietary and biomarker assessments were part of MAGIC, for this study we assessed participant demographics, health behaviors, clinical status, psychological status, and therapeutics. The cohort has been followed annually for up to 4 years after the baseline study enrollment, but herein we present cross-sectional data from enrollment.^15^

Research ethics approval was obtained for all 15 sites involved in the study from their respective research ethics boards. For this report, data are available from 14 centers, as the 15th had enrolled only 1 participant. The study was first registered on April 27, 2017, (ClinicalTrials.gov Identifier: NCT03131414) and was last updated March 16, 2022.

Participants were recruited by convenience sampling. Participants’ age, gender, place of birth, race, highest level of education attained, marital status, and disease duration were all recorded (Table 1). Phenotyping was done according to the Montreal Classification through chart review by trained research coordinators.^16^ Phenotype was recorded at the time of enrollment and not at the time of diagnosis. Behavior of disease is categorized as B1 (inflammatory), B2 (fibrostenosing disease), and B3 (penetrating disease). Location of disease is described as L1 (ileum), L2 (colon only), and L3 (ileocolonic disease). Any L4 disease was used to refer to upper gastrointestinal disease that may accompany any of L1, L2, or L3; perineal disease was also counted separately from other locations or behavior.

**Table 1.** Description of CD cohort.

	All CD	B1	B2	B3	P
	N = 2112	N = 1065 (56.0%)	N = 473 (24.9%)	N = 365 (19.2%)	
Female (%)	59%	61%	59%	57%	0.49
Age (median and IQ)	43 (32-56)	40 (29-54)	51 (37-59)	44 (33-55)	<.0001
White (%)	90.0%	88%	90%	95%	0.0057
Born in Canada (%)	90.6%	90%	90%	94%	0.14
**Marital Status**					
Married or cohabiting	63.6%	61%	68%	66%	0.0014
Widowed	1.9%	2%	2%	2%	
Divorced/separated	9.1%	8%	11%	8%	
Single never married	25.4%	30%	18%	24%	
**Education**					
Grade XII or less	20.3%	20%	20%	20%	0.49
Some college or trade school	37.5%	36%	39%	41%	
College degree or more	42.2%	45%	41%	39%	
Disease duration (yrs ± SD)	15.6 ± 11.8	12.2 ± 10.1	19.4 ± 12.9	18.9 ± 11.8	<.0001
**Location of disease**					
L1	34.9%	31%	47%	32%	<.0001
L2	18.4%	25%	6%	12%	
L3	46.7%	44%	48%	56%	
Perineal disease	15.9%	11%	20%	24%	<.0001
Age at menarche (median and IQ)	13 (12-14)	13 (12-14)	13 (12-14)	13 (12-14)	0.52
GAD-7 score (median and IQ)	3 (0-6)	3 (1-6)	3 (0-7)	3 (0-7)	0.78
PHQ-9 score (median and IQ)	5 (2-8)	5 (2-9)	5 (2-9)	4 (2-8)	0.23

Chi-square tests compared dichotomous and class data. Kruskal-Wallis tests compared continuous data.

Abbreviations: CD, Crohn’s disease; B1, inflammatory; B2, fibrostenosing; B3, fistulizing disease; L1, small bowel disease only; L2, colonic disease only; L3, ileocolonic disease; GAD-7, Generalized Anxiety Disorder-7; PHQ-9, Patient Health Questionnaire-9.

Participants completed a questionnaire to obtain age, gender identity, education level attained, race, ethnic heritage, smoking/alcohol/drug history, comorbidities, medication and therapies, and menstrual status, if relevant, at baseline. For IBD-specific medications, we included azathioprine/6mercaptopurine, methotrexate, tumor necrosis factor inhibitors (TNFi), vedolizumab, ustekinumab, prednisone and other corticosteroids, and mesalamine. At the time of study initiation, we included categories of male, female, and other for gender identity, so we do not have more nuanced data on nonbinary individuals. Participants completed a number of validated clinical measures to assess disease activity, quality of life, physical pain, lifestyle factors, and diet. To assess psychological status, the Generalized Anxiety Disorder-7 (GAD-7)^17^ and Patient Health Questionnaire-9 (PHQ-9)^18^ scores were used to assess symptoms of anxiety and depression, respectively.

Comorbidities and surgeries, both IBD and non-IBD, were self-reported, with common comorbidities identified through a list provided to participants which included depression, eczema, hay fever, migraines, hypertension, hypercholesterolemia, allergic rhinitis, venous thromboembolic disease, rheumatoid arthritis, pyoderma gangrenosum, and chronic fatigue (Supplementary Table 1). All IBD-related surgeries (Supplemental Table 2) as well as cholecystectomy, appendectomy, and comorbidities were verified through chart review.

Finally, medications, complementary and alternative therapies, and diet information was obtained through participant self-reporting utilizing a list of common items for each of these aspects.

## Data Management

Questionnaires at enrollment were completed electronically in the REDCap platform and stored at a central database collection center, the Population Health Research Institute (PHRI), at McMaster University. Study staff reviewed surveys within 2 weeks of receipt and highlighted any missing information. These were then reviewed with the PI, site lead, and study team. The staff contacted participants up to 3 times by phone, email, or in person at a regular clinic visit to remind them to complete questionnaires and to address any missing items.

## Analysis

We evaluated the association between phenotypes and comorbidities in adult CD participants. We also assessed and compared the use of prescription drugs for IBD and for mental health, the use of complementary and alternative therapies (CATs), specific diets, and substance use patterns, including smoking, alcohol, and marijuana/hashish. The χ^2^ test was used to compare characteristics of 3 behavior types (B1, B2 and B3) and also for the locations of L1, L2, and L3. Continuous variables were skewed and described by median and range. Groups (by behavior and location phenotype) were compared using the Kruskal-Wallis test. Multivariate regression analysis for factors associated with IBD-related surgery and separately for non-IBD surgery (eg, cholecystectomy, appendectomy) included disease duration, age, gender, behavior type, and location of disease and produced odds ratios reflecting the relative risk of surgery associated with the factors included in the models. Disease comorbidities were not included in the regression models. A *P* value <0.05 was considered significant. Data analysis in each province was conducted in SAS Version 9.4 (Cary, NC).

## Results

There were 2112 adults with CD enrolled (Table 1). Females made up 59%, and the overall mean age was 44.1 (+/-14.9 SD) years. The majority were white (90.7%), born in Canada (90.4%), and married or cohabiting (63.6%). A majority had at least a college degree (62.2%), while 20.2% had grade 12 or less education. Based on Montreal Classification, half the sample had inflammatory disease behavior (B1 = 1065; 50.4%), close to one-quarter had fibrostenosing disease (B2 = 473; 22.4%), and the remainder had penetrating disease (B3 = 365; 17.3%), with 9.9% missing this information (*n* = 209). Perianal disease was identified for 14.2% (*n* = 302) participants. Pertaining to disease location, 35.2% of patients had disease in L1, 16.8% in L2, and 48% in L3. Only 9 (0.4%) patients had L4 disease.

There was no difference in phenotype by gender. White patients were significantly more likely to have B2/B3 disease (*P* = .003), but there was no difference in the occurrence of perianal disease (*P* = .64). Median age at menarche was 13 (range 9, 19), and this was not different by phenotype. There was no difference in anxiety (GAD-7) or depression (PHQ-9) scores by phenotype, whether those scores were analyzed in a linear or binary (with a cut point for active at 11) fashion. Disease duration was significantly different depending on disease behavior type (B1 = 12.2 ± 10.1; B2 = 19.4 ± 12.9; B3 = 18.9 ± 11.8, *P* < .0001) but was generally long enough for final phenotype to be established. Disease location was significantly different by behavior type (*P* < .0001). Isolated colonic disease was much less likely to be fibrostenotic or penetrating than inflammatory disease. Penetrating disease was more likely to be associated with ileocolonic location than other locations.

There were differences in phenotype by year of diagnosis (Table 2). In the later years of the study, those newly diagnosed were less likely to have ileocolonic disease (L3) than L1 or L2, and B1 disease was significantly more common than B2 and B3.

**Table 2.** Distribution of phenotype by era of diagnosis.

Year of diagnosis	1959-1999	2000-2010	2011-2022	*P*
**Location %**				
N	646	679	694	
L1 ileum only	34	32	38	0.016
L2 colon only	13	20	20	
L3 ileocolon	53	48	42	
**Behavior %**				
N	581	614	655	
B1	37	57	71	<0.0001
B2	36	23	18	
B3	27	20	11	

Perineal disease was most commonly seen in persons with B3 disease (24%) than other behaviors (B1 11%; B2 20% disease, *P* < .0001; Supplemental Table 3). It was also more likely to be seen in those with ileocolonic disease (L3;19%) vs L2 disease (17%) and L1 disease (11%; *P* < .0001). Only 9 (0.4%) were reported to have L4 disease. Of those with both B3 and L3 disease, 27% had perineal disease.

### Medication Use by Phenotype Behavior (Table 3) *Biologics*

The majority had some experience with an anti-TNF, with 64.2% reporting use of this type of medication at some point, and differences were found between disease behavior subgroups (B2 > B1; B3 > B1, *P* < .0001); 15.4% had used ustekinumab (B2 > B3 > B1 *P* = .0002), and 9.8% had experience with vedolizumab (no difference between B1, B2 or B3). Use of one biologic was most common in those with B3 disease (57%). Those with B2 were most likely to have used 2 biologics (19%), and only 4% of those with B3 disease had used all of anti-TNF, ustekinumab, and vedolizumab.

**Table 3.** Prescription medical therapy and health habits by behavior phenotype (all data presented are %).

Use		B1	B2	B3	*P*
**Anti-TNF**					
	Never	42	31	27	
	Previously	15	23	22	
	Currently	43	47	51	0.01
	Ever used	58	69	73	<0.0001
**Vedolizumab**					
	Never	90	89	92	
	Previously	3	4	2	
	Currently	7	7	6	0.66
	Ever used	10	11	8	0.46
**Ustekinumab**					
	Never	88	78	84	
	Previously	3	4	5	
	Currently	10	18	11	0.08
	Ever used	12	22	16	0.01
**Currently using any biologic**					
		57	66	65	0.0008
**Number ever used**					
None		37	26	25	<0.0001
1		52	56	61	
2		9	15	12	
all		2	3	3	
Ever used thiopurines		59%	71%	71%	<.0001
Ever used methotrexate		25%	33%	32%	0.0011
Ever used 5-ASA		53%	58%	57%	0.26
Ever used corticosteroids		71%	79%	81%	0.0005
Ever cigarette smokers		38%	50%	43%	0.0017
**Amongst ever smokers**					
Quit smoking		77%	78%	72%	0.48
Still smoking		23%	22%	28%	
Ever used marijuana or hashish (M/H)		63%	68%	65%	0.27
**Patterns of M/H in the past year**					
None		47%	53%	46%	**0.012**
Some		42%	38%	35%	
Daily		10%	10%	19%	
**Alcohol use**					
Never		18%	15%	suppressed	**0.014**
Some		59%	55%		
2-6x/wk		20%	26%		
Daily		2%	4%		

#### Standard therapies

Additionally, 64.3% had used thiopurines (B2, B3 > B1, *P* = .001), 28.0% had used methotrexate (B2, B3 > B1 *P* = .001), just over half had used 5-ASA (54.9%; no difference between B1, B2, or B3), and three-quarters had used oral steroids at some point in their IBD care (74.7%; B3 > B2 > B1 *P* = .0005). Prescription psychiatric drugs use was reported as “never” in 72%, “ever” in 28%, and “ongoing” in 19%. Two hundred twenty-one of 1518 who responded (15%) reported current 5-ASA use. This was associated with similar use of thiopurines (18% in 5-ASA users vs 21% in non-5-ASA users; *P* = .24) and methotrexate (5% in 5-ASA users vs 8% in 5-ASA nonusers; *P* = .10) but there was a lower use of anti-TNF (24% in 5-ASA users vs 48% in 5-ASA non users; *P* < .0001).

Those with more remote diagnoses for CD, between 1959 and 1999, were significantly more likely to be currently receiving prednisone (17%, *P* = .0036) or 5-ASA (19%, *P* = .001) compared with those diagnosed in more recent decades of 2000 to 2010 (11% and 13%, respectively) or 2011 to 2022 (12% and 12%, respectively). Persons diagnosed between 1959 and 1999 were more likely to be currently using ustekinumab (14%) compared with those diagnosed from 2000 to 2010 (12%) and those diagnosed from 2011 to 2022 (9%; *P* = .0183). However, anti-TNF was least likely to be used by persons diagnosed between 1959 and 1999 (39%, *P* = .0003; Supplemental Table 4).

### Health Habits (Table 3)

Regarding substance use, 42.1% reported cigarette smoking at some point in their life. The vast majority of smokers had quit (76.4%), and 23.6% indicated they are still smoking. Persons with B2 disease (49.6%) were more likely to have been smokers at some point compared with those with B3 disease (42.7%) or those with B1 disease (38.4%; *P* = .0017). There was no difference by disease behavior regarding current smoking. Those with isolated ileal disease (L1) were more likely to be “ever” smokers (47%, vs 42% for L2, 39% for L3, *P* = .014; Supplemental Table 5).

Marijuana or hashish (M/H) was tried by 64.9% at some point in their life. Looking at patterns of use of these substances in the past year, 48.3% reported no use, 40.1% reported some use, and 11.6% reported daily use. Alcohol use was reported as “never” in 16.7%, some use in “59.5%,” 2-6 times per week in 21.2%, and daily use in 2.5%. There was no difference by disease behavior or disease location for pattern of marijuana, hashish, or alcohol use.

### Diet and Complementary and Alternative Therapies

We inquired about the use of 2 supplements (psyllium and unspecified fiber), 3 diets (gluten-free, low FODMAP and paleolithic), and alternate therapy (Tables 4 and 5). With regards to specific diet or use of CATs, one-third used some type of CAT/supplement or diet. The most common diet used was gluten-free, used by 6% in the past and 7.9% currently. The most common supplement used was psyllium, used by 7.4% in the past and 4.3% currently (Tables 4 and 5). Although the differences are not significant, 2.6% of B2 are current users of a fiber supplement compared with 3.8% of B1 and 3.1% of B3. The most highly different use of an alternative therapy by disease behavior was the use of acupuncture in 9% of those with B3 disease, which was more than in the other groups (Tables 4 and 5). Of those using CAT or a specific diet, 4 or more of CAT or diet were used by 5.9%, 3 by 5.2%, 2 by 8.5%, and 1 by 13.8%. There was a very modest difference in diet or CAT use comparing disease behavior type, with slightly more use of acupuncture by those with B3 (5.95%) than B2 (4.8%) or B1 (2.9%; *P* = .042).

**Table 4.** Diet, food supplements and complementary and alternative therapy (CAT). 4a) Percent current and percent past use.

Diet and Alternate Therapies	N	% Current Use	% Past Use
Psyllium	2036	4.3	7.4
Fiber (unspecified)	2036	3.6	4.2
Gluten free diet	2036	7.9	6
Low FODMAP	2032	3.8	4.7
Paleo diet	2034	1.8	1.8
Herbal remedy	2035	3.8	2.5
Acupuncture	2036	2.3	4.1
Homeopathy	2035	1.1	1.7
Naturopathy	2036	3.2	3.6
Osteopathy	2035	1.8	1.2
Other non-medical therapy	2034	5.2	0.9

**Table 5.** Comparison of diet, food supplement and CAT use by behavior phenotype.

Diet and Alternate Therapies	% Ever Used				*P*
	All	B1	B2	B3	
Psyllium	11.6	11	11	14	0.41
Fiber (unspecified)	7.8	8	8	8	0.94
Gluten free diet	13.9	14	14	12	0.55
Low FODMAP	8.5	8	9	9	0.89
Paleo diet	3.5	3	4	3	0.34
Herbal remedy	6.3	6	6	8	0.23
Acupuncture	6.3	5	7	9	0.011
Homeopathy	2.8	2	3	5	0.057
Naturopathy	6.8	7	6	6	0.83
Osteopathy	3.0	2	5	3	0.046
Other non-medical therapy	6.2	6	5	7	0.56
Number					0.32
None	66.6	68	64	66	
1	13.8	13	17	13	
2	8.5	8	8	9	
3	5.2	6	4	5	
4	3.0	2	3	5	
5+	3.0	3	3	3	

Of all respondents who answered all 5 questions, 67% reported no use, and 78% reported no current use of supplements, CAT, or diets. Nineteen percent reported using 2 or more supplements, diets, or alternative therapy at some time, and 9% used 2 or more currently. There was a high correlation between supplements and correlations among all therapies. For current use, psyllium was associated with the paleolithic diet and fiber supplement with the gluten-free diet (GFD). People were more likely to use any diet if they were also on another diet and more likely to use an alternate therapy if using another. All 3 health-seeking behaviors were correlated. (Supplementary Tables 6, 7, 8).

### Surgery Comparisons by Phenotype Behaviors (Supplemental Table 9 and Table 6)

Surgery related to IBD occurred across each of the disease behavior types of B1, B2, and B3 at rates of 23%, 64%, and 74%, respectively. Rates of IBD-related surgery by location of disease were 48% in L1, 21% in L2, and 51% in L3. The variables more highly associated for IBD-related surgery, on multivariate logistic regression, included disease behavior (B2: OR, 3.80; 95% CI, 2.92-4.93; B3: OR, 7.71; 95% CI, 5.69-10.45) and disease duration. Age did not affect surgery risk in the model. The L1 location has a similar surgery risk as L3 (OR, 0.90; 95% CI, 0.67-1.19; *P* = .44). The L2 location (colon only) has a much lower likelihood of surgery (OR, 0.19; 95% CI, 0.12-0.30; *P* < .0001). Considering other surgeries, cholecystectomies were more common in persons with B2 disease, although this was not statistically significant in the multivariate logistic model (OR, 1.27; 95% CI, 0.97-2.32). In a multivariate model, the variables associated with having cholecystectomy were L1 location (OR, 1.64; 95% CI, 1.09-2.47) and age. Appendectomies were more common in B2 (17%, OR, 1.81; 95% CI, 1.27-2.56) and B3 (24%, OR, 3.12; 95% CI, 2.21-4.43) than B1 (8%).

**Table 6.** Multivariate logistic regression analysis of factors associated with surgery [odds ratios (OR) and 95% CI].

Predictor	IBD-related Surgeries^**^	Cholecystectomy	Appendectomy
Total N^*^	1849	1849	1849
Events	805	130	248
Behavior status B1	ref		
B2	3.80 (2.92-4.93)	1.50 (0.97-2.32)	1.81 (1.27-2.56)
B3	7.71 (5.69-10.45)	1.27 (0.77-2.1)	3.12 (2.21-4.43)
Location L3	ref		
L1	1.00 (0.78-1.28)	1.64 (1.09-2.47)	1.15 (0.85-1.55)
L2	0.24 (0.16-0.35)	1.36 (0.78-2.35)	0.84 (0.54-1.30)
Age: 18-39	ref		
40-59	1.08 (0.82-1.41)	2.52 (1.48-4.29)	1.95 (1.35-2.81)
60+	1.19 (0.84-1.68)	5.35 (3.07-9.33)	2.34 (1.53-3.58)
Duration of IBD (yrs) <10	ref		
10-24	2.95 (2.28-3.81)	1.20 (0.77-1.89)	1.13 (0.79-1.60)
25+	9.28 (6.47-13.3)	1.17 (0.7-1.97)	1.61 (1.08-2.41)

^*^Only 1849 of 2112 participants reported outcomes for these surgeries.

^**^The data reflect 1 or more surgeries vs none (ie, a participant could have several IBD-related surgeries but is only counted once).

### Comorbidities by Phenotype Behaviors

The 5 most common self-reported comorbidities were depression (14.1%), eczema (9.9%), hay fever (9.7%), migraines (9.5%), and hypertension (9.4%). Persons with B1 were more likely to have hypercholesterolemia (6% vs 3% for B2 and B3; *P* = .0018) and allergic rhinitis (5% vs 3%; *P* = .016). Venous thromboembolic disease was less common in B1 (1% vs 2%; *P* = .03). Rheumatoid arthritis (8% vs 5%; *P* = .031), pyoderma gangrenosum (1% vs 0.3%; *P* = .042), and chronic fatigue (7% vs 4%; *P* = .035) were more common in those with perineal disease. Hypercholesterolemia (5% vs 2% *P* = .03) was more common in those without perineal disease.

## Discussion

In this large national Canadian sample of over 2100 adults with CD, approximately half had inflammatory disease. Persons with complex disease (ie, B2 or B3 behavior) had significantly longer disease duration, so it is possible that some of those with B1 disease may subsequently present with a stricture or fistula complication. Nonetheless, the disease duration in this sample was sufficiently long even for those with B1 disease, given the average duration of nearly 16 years, to suggest that final disease behavior has been reached for most. The majority of the cohort was white, which is consistent with US population studies.^19^ It was noted that white patients were more likely to have complicated disease (B2/B3 disease). This finding will need to be replicated with a larger sample of patients from other races with IBD.

### Surgeries

In this contemporary era, two-thirds to three-quarters of persons with complicated disease had IBD surgery. Uncomplicated or inflammatory disease was less evident in those with appendectomy. This is noteworthy since persons with ulcerative colitis are typically less likely to have appendectomy.^20^ Hence, whatever leads to appendicitis, if the affected individual has CD perhaps this risk factor for appendicitis^21^ is associated with more transmural disease and complicated intestinal disease. Cholecystectomy is more common in those with ileal disease, which is not surprising considering what is known about enterohepatic bile salt circulation.^22^ Furthermore, hypercholesterolemia was more common in those with a less aggressive form of CD such as B1 (inflammatory disease) and in persons without perineal disease. An association between hypercholesterolemia and CD was previously reported; however, a specific CD phenotype association was not studied.^23^ Other comorbidities may be associated with specific CD phenotypes. This requires further study to elucidate possible pathophysiologic correlates.

### Medical Therapy

Two-thirds of persons with B2 or B3 disease have used anti-TNF or thiopurines, which were both significantly more than in persons with inflammatory disease. Further, corticosteroids were used in three-quarters of persons and were more likely to have been used in persons with more complicated disease. Over half of persons with CD used 5-ASA, including those with complicated disease. Hence, despite advances in biological therapies, corticosteroids are still a commonly prescribed component of our therapeutic armamentarium. However, the use of 5-ASA in over half of persons with CD requires further exploration. For instance, it is important to know whether these drugs are being prescribed before these persons consult with a gastroenterologist. Whether it is primary care, surgery, or gastroenterology, there must be greater education undertaken to ensure that persons who have more than inflammatory colonic disease are being treated with other, more effective medications than 5-ASA.

### Supplements/Diet/Complementary and Alternative Therapies

One-third used some type of supplement, diet, or CAT. The US National Center for Complementary and Integrative Health reported that 30% of adults use some form of CAT.^24^ In IBD clinic-based studies, CAT was reported to be used by 44% in Israel,^25^ 48% in Sweden,^26^ and 45% in Australia.^27^ In a German study of members of an IBD support group, approximately half used CAT at some point.^28^ We did not inquire about all possible dietary regimens, and so we have not captured other common ones used by persons with IBD. We inquired about 3 diets that were not designed for persons with IBD. No diets have been uniformly shown to benefit persons with IBD,^29^ although some diets have been designed specifically for persons with IBD.^30^ In our study, gluten-free diets were used by nearly 8% of respondents, even though they did not have celiac disease. A US study of over 1600 persons with IBD enrolled though a patient support group also found that 8% of persons with IBD currently used a GFD.^31^ Nearly 5% of a Swiss IBD cohort were using a gluten-free diet.^32^ In our study, nearly 4% used a low FODMAP diet, and nearly 2% used a paleolithic diet. Not surprisingly, users of these special diets were significantly more likely to use supplements or CAT. Of those using CAT or a specific diet, approximately 6% to 9% used 2 to 4 different approaches. One was used by nearly 14%. While we did not ask why supplements/diet/CAT was being used, in a previous study from Manitoba, Canada, it was shown that 14% used CAT consistently over a 5-year time span; and among users only, 1 in 5 used it for their IBD specifically.^33^

Some aspects of our data are similar to that reported in a large Swiss cohort. The Swiss Inflammatory Bowel Disease Cohort Study (SIBDCS) was launched in 2006, and at last published follow-up in 2019, there were 2011 persons with CD with approximately 15 years’ disease duration. The distribution of disease behavior was very similar to our Canadian cohort: 55% had inflammatory disease, 40% fibrostenosing, and 15% penetrating.^34^ However, the Swiss cohort had a much higher level of perianal disease at 36%. Further, 32.5% had isolated colonic disease compared with only 17% in our Canadian cohort. It was also reported elsewhere that 39.6% of Swiss CD patients were active smokers, which raises an interesting question about whether smoking is at all explanatory for the difference in perianal disease prevalence compared with Canadians with CD. Also of note, 60% of Swiss patients had been prescribed 5-ASA, suggesting that excessive use of 5-ASA is not just occurring in Canada. Regarding diet in the Swiss cohort, 4.8% reported a vegetarian diet and 4.6% followed a gluten-free diet,^34^ the latter is slightly lower than the prevalence of gluten-free diet use by Canadians with CD. In an assessment of phenotype associations in the Swiss cohort on multivariate analysis, being male, smoking, colonic involvement of CD, and stenosis were all associated with perianal fistulas. Disease duration, colonic CD, and stenosis were independently associated with the occurrence of nonperianal fistulas.^35^ In our Canadian cohort, persons with B3 and L3 disease had a high prevalence of perineal disease at 27%.

The European Epi-IBD inception cohort was also similar to our Canadian cohort. Of the 488 persons enrolled, 71% had inflammatory disease and by 5 years follow-up 61% had inflammatory disease; hence 10% progressed to more complicated disease.^36^ At enrollment, 32% had L1 disease, 32% had L2 disease, 29% had L3 disease, and 6% had isolated L4 disease. By 5 years, 12% had a change in disease location. By 5 years, 14% had perianal disease, and the majority had inflammatory (B1) and colonic disease. There were no statistically significant predictors of a change in disease behavior; however, those with either fibrostenosing or penetrating disease were more likely to have progression of disease location. The main predictors of having surgery were having either fibrostenosing or penetrating disease and not disease location, age, gender, or smoking status. The frequency of use of 5-ASA was 62%, corticosteroids was at least 60% (some used budesonide), immunomodulators use was 64%, and biologicals use which was mostly TNFi was 30% (more than twice as high in western than eastern Europe). This considerably lower rate of TNFi use than in our Canadian cohort reflects the enrollment period for this study of 2010. Smoking was not an outcome predictor in this study, much like in our Canadian study. This may suggest that in more recent years in the west, smoking may not have as obvious a role in disease outcomes.

In a large population-based study of 1647 incident cases of CD in 2017 in Spain, 55% had L1, 19% L2, and 26% L3 disease.^37^ In our study, we had a much higher proportion of persons with L3 disease and lower with L1 disease. In Spain, 82% had inflammatory disease, while 11% had fibrostenosing disease, 7% had penetrating disease, and 11.2% had perianal disease. Although this is a much higher prevalence of inflammatory CD than we reported, this report was of the first year of disease. Hence, it is not yet known what proportion of Spaniards with CD will still have inflammatory disease only after 10 years of disease. In this cohort, 38% were former smokers, although current smoking was not reported.

In assessing European cohorts with enrollment over decades, more recent cohorts are more likely to have predominantly inflammatory disease. In Orebro, Sweden, 472 persons were diagnosed with CD from 1963 to 2005.^38^ The proportion of patients with complicated disease behavior 5 years after diagnosis decreased from 54.4% in those diagnosed 1963 to 1975 to 44% in those diagnosed 1976 to 1990 to 33.3% in patients diagnosed 1991 to 2005; the proportion of patients progressing to complicated disease behavior was stable among those with nonstricturing, nonpenetrating disease. Hence from 1991 to 2005, 73.4% had B1 disease at diagnosis, 12.8% had B2 disease, and 13.8% had B3 disease. By 5 years, 67% had B1 disease and 33% had B2/B3 disease, so only 6.4% progressed. Perineal disease was evident in 8.4%. For those diagnosed from 1991 to 2005, the disease location distribution was L1 (39.9%), L2 (35.8%), and L3 (26.6%), and only 1.8% had L4 disease (38).

In Veszprem, Hungary, 946 persons were diagnosed with CD between 1977 and 2018. The cohort was divided into 3 cohorts by era: cohort A (1977-1995), cohort B (1996-2008), and cohort C (2009-2018).^39^ The cumulative probability of disease behavior progression in patients with inflammatory (B1) behavior at diagnosis into fibrostenosing or penetrating phenotype (B2/B3) was 18.4 ± 1.7% after 5 years, 27.9 ± 2.0% after 10 years, and 36.7 ± 2.5% after 20 years. However, the probability of disease behavior progression from inflammatory (B1) to fibrostenosing or penetrating (B2/B3) phenotype significantly decreased (27.1 ± 5.3%/ 21.5% ± 2.5%/11.3% ± 2.2% in cohorts A/B/C after 5 years, 44.3 ± 5.9%/ 30.6 ± 2.8%/ 16.1% ± 2.9% after 10 years; *P* < .001).

However, in a cohort study from Holland, the progression over 5 years to a complicated phenotype was 21% in both persons diagnosed in the early 1990s, as well as in persons diagnosed from 2006 to 2011.^40^ The cumulative 5-year probability of having complicated disease was 35.5%. This reached approximately 55% by 20 years suggesting more disease progression than we are reporting from Canada and elsewhere in Europe.

The increasing prevalence of inflammatory disease may reflect earlier diagnosis enhanced by newer technologies such as capsule endoscopy or magnetic resonance enterography. However, in the face of plateaued incidence of disease in the west, the low progression rate to more complicated disease over time may either reflect enhanced management with better drugs or a true change in phenotype.

Our Canadian data are not that dissimilar to data from Asia, as noted in the Introduction. However, there was a considerable spectrum regarding disease behavior in Asian and western populations. In Asian countries, by 10 years approximately 50% have complicated disease.^9^ In a recent study from Japan of 673 newly diagnosed adults with CD, 63% had an inflammatory phenotype; 60% had ileocolonic disease; and 49% had perianal lesions, reflecting similar disease behavior and location compared with our data, albeit a much higher rate of perineal disease.^10^ In other Asian studies, rates of inflammatory disease were also high at 66% to 81%.^7,8^

Our study has several strengths, including the advantage of in-depth chart review to categorize patient phenotype and comorbidities and well-established disease in this cohort, suggesting that final disease phenotype has likely been reached for many. Further, our study is multicentered and extends from coast to coast in Canada. One limitation is a marked majority of participants are white and born in Canada; hence, it will be of interest to determine if some of the CD phenotype associations are replicated in more diverse IBD samples, especially other western populations and immigrant populations in western countries. Also, in terms of understanding the relationship between surgeries and different IBD phenotypes, we used statistical approaches for predictive risk; however, we do not know the temporal relationship of these surgeries and the IBD disease behavior or location given the cross-sectional design of the study.

By exploring this large contemporary CD cohort, we have determined that inflammatory disease is the main CD phenotype in Canada and that in the past 10 to 20 years CD-related surgery remains very common. Despite its known toxicity, use of corticosteroids is a common treatment along with nontoxic but questionably effective 5-ASA. We have identified associations such as appendectomy and venous thromboembolic disease with more complicated disease and found perianal disease to be associated with more disease comorbidities. Cholecystectomy was associated with ileal disease. All of these associations require confirmation in other cohorts as well as physiologic explanations. Perhaps uncovering the nature of these associations may identify mechanisms for the development of certain phenotypes of CD.

## Supplementary Data

Supplementary data is available at *Inflammatory Bowel Diseases* online.

izae055_suppl_Supplementary_Tables_1-9
