# Supplementary material for: Crohn’s Disease Phenotypes and Associations With Comorbidities, Surgery Risk, Medications and Nonmedication Approaches: The MAGIC in IMAGINE Study
Source: Inflamm Bowel Dis. 2024 Mar 27;31(1):113–22. doi: 10.1093/ibd/izae055 (PMC11700885; doi:10.1093/ibd/izae055)
Supplement: izae055_suppl_Supplementary_Tables_1-9 [file izae055_suppl_supplementary_tables_1-9.docx]

**Supplemental Tables**

**Supplemental Table 1.** **List of other diseases asked in survey**

rheumatoid arthritis

ankylosing spondylitis / sacroiliitis

iritis / uveitis

primary sclerosing cholangitis

erythema nodosum

pyoderma gangrenosum

eczema

atopic dermatitis

psoriasis

asthma

allergic rhinitis

hay fever

multiple sclerosis

celiac disease

acute gastroenteritis

diabetes

ischemic heath disease

stroke

COPD

cirrhosis

chronic kidney disease

hypertension

hypercholesterolemia

depression

osteoporosis

venous thromboembolic disease

interstitial cystitis

vulvodynia

migraines

chronic fatigue

fibromyalgia

Other

**Supplemental Table 2. List of IBD-specific surgeries**

Bowel resection

Colostomy

Pelvic pouch

Stricturoplasty

Fistulectomy

Incising and draining of abscess related to fistula

**Supplemental Table 3. Prevalence of perianal disease by disease phenotype**

|  | %perianal | P value |
| --- | --- | --- |
| L1 ileum/small bowel | 11 | <0.0001 |
| L2 colon only | 17 |  |
| L3 ileocolon | 19 |  |
|  |  |  |
| B1 inflammatory | 11 | <0.0001 |
| B2 fibrostenosing | 20 |  |
| B3 penetrating | 24 |  |
|  |  |  |
| L1 ileum B1 | 7 | 0.032 |
| B2 | 15 |  |
| B3 | 12 |  |
|  |  |  |
| L2 Colon B1 | 10 | <0.0001 |
| B2 | 38 |  |
| B3 | 43 |  |
|  |  |  |
| L3 ileocolon B1 | 15 | 0.0002 |
| B2 | 22 |  |
| B3 | 27 |  |

**Supplemental Table 4: Current users (%) of specific medications by era of diagnosis**

|  | 1959-1999 | 2000-2010 | 2011-2022 | P value |
| --- | --- | --- | --- | --- |
| Azathioprine/6MP | 20 | 21 | 21 | 0.58 |
| Methotrexate | 7 | 6 | 11 | 0.028 |
| Anti-TNF | 39 | 51 | 47 | 0.0003 |
| Vedolizumab | 6 | 7 | 7 | 0.38 |
| Ustekinumab | 14 | 12 | 9 | 0.018 |
| Prednisone | 17 | 11 | 12 | 0.004 |
| 5ASA | 19 | 13 | 12 | 0.001 |

**Supplemental Table 5. Lifestyle habits by disease location**

|  | L1 Ileum | L2 Colon | L3 Ileocolonic | P |
| --- | --- | --- | --- | --- |
|  |  |  |  |  |
| Ever cigarette smokers | 47% | 42% | 39% | 0.014 |
| N | 535 | 275 | 735 |  |
| Amongst ever smokers |  |  |  |  |
| quit | 75% | 82% | 76% | 0.30 |
| still smoking | 25% | 18% | 24% |  |
| N | 245 | 111 | 279 |  |
| Ever used marijuana or hashish (M/H) | 64% | 61% | 67% | 0.12 |
| N | 537 | 277 | 738 |  |
| Patterns of M/H past year |  |  |  |  |
| none | 53% | 49% | 46% | 0.25 |
| some | 36% | 41% | 42% |  |
| daily | 11% | 10% | 13% |  |
| N | 341 | 168 | 494 |  |
| Alcohol use |  |  |  |  |
| never | 18% | 17% | 16% | 0.44 |
| some | 59% | 58% | 60% |  |
| 2-6x/wk | 20% | 22% | 22% |  |
| daily | 3% | 3% | 2% |  |
| N | 539 | 277 | 743 |  |

**Supplementary Table 6. Relationships among diet and alternate therapy. Comparison of the “never used” group with subjects reporting use currently or in the past. Pearson correlation coefficients above and probability below.**

**Supplementary Table 7**. **Relationships among diet and alternate therapy. Comparison of the current user group with subjects reporting no current use. Pearson correlation coefficients above and probability below.**

**Supplementary Table 8. Relationships among use of any diet and any alternate therapy. Comparison of the current user group with subjects reporting no current use. Pearson correlation coefficients above and probability below.**

**Supplemental Table 9. IBD-related surgeries, cholecystectomies and appendectomies by phenotype and demographics**

| % with Surgery | |  |  |
| --- | --- | --- | --- |
|  | IBD related surgeries | Cholecystectomy | Appendectomy |
| Behavior status | |  |  |
| B1 | 23 | 5 | 8 |
| B2 | 64 | 10 | 17 |
| B3 | 75 | 8 | 24 |
| P | <.0001 | 0.0049 | <.0001 |
| Location |  |  |  |
| L3 | 48 | 10 | 15 |
| L1 | 18 | 7 | 10 |
| L2 | 49 | 5 | 13 |
| P | <.0001 | 0.0049 | 0.045 |
| Age: |  |  |  |
| 18-39 | 33 | 3 | 7 |
| 40-59 | 50 | 8 | 17 |
| 60+ | 55 | 16 | 20 |
| P | <.0001 | <.0001 | <.0001 |
| Duration of IBD (yrs) | |  |  |
| <10 | 22 | 5 | 9 |
| 10-24 | 47 | 7 | 13 |
| 25+ | 77 | 11 | 24 |
| P | <.0001 | 0.0026 | <.0001 |
